# Psychiatric symptoms and behavioral adjustment during the COVID-19 pandemic: evidence from two population-representative cohorts

**DOI:** 10.1038/s41398-021-01279-w

**Published:** 2021-03-17

**Authors:** Wai Kai Hou, Tatia Mei-chun Lee, Li Liang, Tsz Wai Li, Huinan Liu, Horace Tong, Menachem Ben-Ezra, Robin Goodwin

**Affiliations:** 1 Department of Psychology, The Education University of Hong Kong, Hong Kong, SAR China; 2 Centre for Psychosocial Health, The Education University of Hong Kong, Hong Kong, SAR China; 3 State Key Laboratory of Brain and Cognitive Sciences, The University of Hong Kong, Hong Kong, SAR China; 4 Laboratory of Neuropsychology & Human Neuroscience, The University of Hong Kong, Hong Kong, SAR China; 5 School of Social Work, Ariel University, Ariel, Israel; 6 Department of Psychology, University of Warwick, Coventry, UK

**Keywords:** Psychiatric disorders, Depression

## Abstract

This study examined prevalences of anxiety and depression and their correlations with daily routines among Hong Kong Chinese during the COVID-19 pandemic. Random digit dialing recruited two population-representative samples of 6029 residents during a period of low infection and limited intervention (survey 1: *n* = 4021) and high incidence and intensive measures (survey 2: *n* = 2008). Prevalence of anxiety for survey 1 and survey 2 were 14.9% and 14% and depression were 19.6% and 15.3%, respectively. Increased odds of anxiety and depression were associated with disrupted routines and lower socioeconomic status in both surveys, whereas depression was inversely related to the novel preventive routine of avoiding going to crowded places in survey 1. The prevalences of anxiety and depression were higher than preceding public health/social crises. A heavier burden of psychiatric conditions was evidenced amongst people experiencing disrupted daily routines across different phases of the pandemic and without novel preventive routines in the early phase.

## Introduction

The COVID-19 pandemic places a considerable burden on populations worldwide and is having a severe economic impact^1^. In response to the pandemic, different forms of lockdown, quarantine, and social/physical distancing are currently being implemented, restricting interactions within communities and countries and across countries and regions. Key changes in life domains, including restrictions on personal mobility (due to home confinement), on interpersonal relationships (through reduced face-to-face interaction), and on occupation/education roles are akin to the functional impairments associated with common mental disorders such as anxiety and depression. This suggests that such measures will have a significant mental health toll^2^, while people will have to live with the pandemic by altering different aspects of their living until a safe, effective, and affordable vaccine is available. Guidelines on behavioral adjustment could cost-effectively reduce the potential burden of mental health need upon already stressed medical care sectors^3^.

Governments^4,5^ and representative non-governmental organizations^6,7^ have disseminated a number of recommendations to increase public awareness of the mental health impact of disrupted daily routines. These recommendations have encouraged people to regularize existing positive routines and create new useful and meaningful activities (e.g., household chores, exercising, leisure activities, and new means of socialization). A recent model suggested that psychological resilience during trauma and chronic stress conditions is largely determined by the regularity of daily routines^8^. Survivors of natural disasters successfully sustain the regularity of daily activities to deal with post-disaster stress^9^, with the restoration and sustainment of pre-disaster daily life associated with decreased psychological distress in the 6 years following the Great East Japan Earthquake^10^. Amongst conflict-affected migrants, the disruptions of different types of daily experiences was associated with higher levels of psychiatric symptoms and general psychological distress^11^.

We quantify the population prevalences of anxiety and depression as the pandemic unfolded and evaluate associations of clinically significant symptoms with different types of daily routines in two population-representative samples in Hong Kong. Our study assessed disruptions to longstanding routines and the addition of novel preventive routines^8,12^. Longstanding routines were parsed into primary routines (i.e., healthy eating and sleep), essential for maintaining livelihood and biological needs, and secondary routines (i.e., socializing and leisure activities) that fulfil personal motivations and preferences^3,8^. Novel preventive measures of enhancing personal hygiene, including wearing a facemask, washing hands in different occasions, and covering mouth when coughing and sneezing, were inversely associated with psychiatric symptoms amongst convenient samples during the outbreak in China^13^. We anticipated that disruptions to primary and secondary routines and the absence of new preventive routines will relate to increased odds of anxiety and depression.

## Materials and methods

### Respondents and procedure

**Fig. 1:**
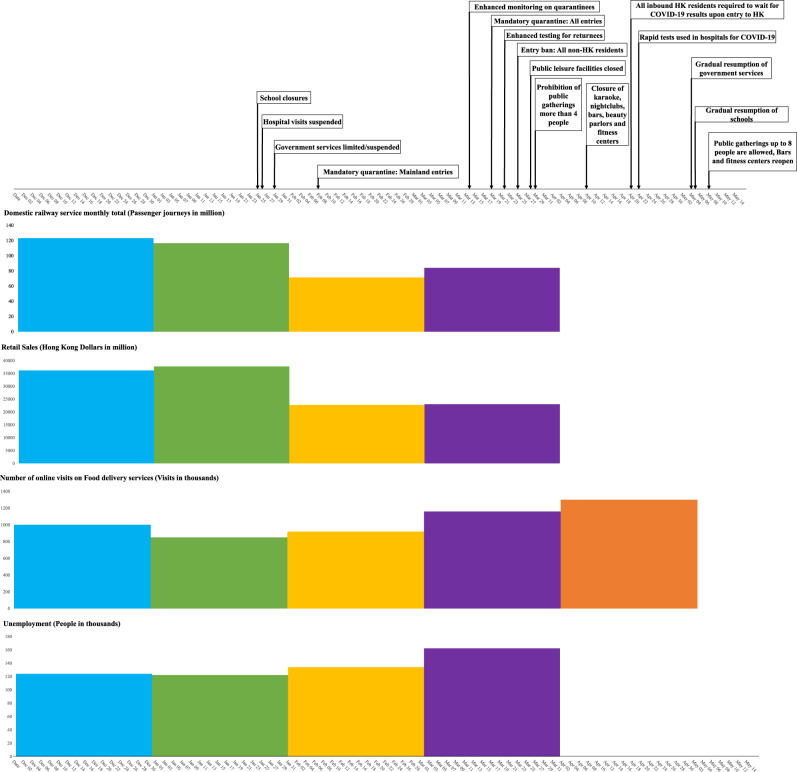
Changes in population behavior between December 2019 and May 2020. This figure illustrates four indicators that reflected changes in population behavior during the COVID-19 pandemic in Hong Kong. Monthly total domestic railway service users were reported by the Mass Transit Railway (MTR) Corporation of Hong Kong, the sole railway provider in Hong Kong. The data represents the monthly total number of passengers. Data on retail sales and unemployment were extracted from the Hong Kong Monthly Digest of Statistics provided by the Census and Statistics Department of the Hong Kong SAR government. Retail sales represents the monthly total retail sales in Hong Kong across all retail outlets, and unemployment represents the total number of unemployed residents in Hong Kong across all industries. The number of visits on food delivering services were extracted from SimilarWeb and the data shows the total number of online visits to Food Panda and Deliveroo, the two major food delivering service providers in Hong Kong. All data were taken as on May 18, 2020.

Following approval from the Ethics Committee of The Education University of Hong Kong, two telephone surveys, with different respondents, were conducted by the Centre for Communication and Public Opinion Survey of the Chinese University of Hong Kong, and Hong Kong Public Opinion Research Institute on February 25–March 19, 2020 (survey 1) and April 15–May 1, 2020 (survey 2). A total of 70 cases of COVID-19 had been reported in Hong Kong up to February 24, a period of low infection and limited public health intervention; 871 new cases were reported between March 15 and April 14, a period of high incidence and intensive control measures (Fig. 1)^14^. The sample size required for a two-tailed test with *α* = 0.05 and power = 0.80 was computed by G*Power^15^. With an odd ratio of 1.89 and a population prevalence of clinically significant psychiatric symptoms of 3% in ordinary time (H_0_ = 0.03)^16,17^, the minimum sample size was 606. A computer-assisted telephone interview (CATI) system was used. Random digit dialing has been shown to produce a population representative sample of Hong Kong residents^18^. A dual-frame sampling approach with both landline and mobile phone numbers (50% each) was utilized. Telephone numbers were randomly extracted from databases released by the Hong Kong Communication Authority. The interviewers received formal training to conduct the telephone surveys. On-site supervision, voice recording, screen captures, and real-time camera surveillance ensured the consistency and quality of the survey data. At least 5% of the completed interviews were randomly drawn and checked. All respondents were (1) Hong Kong Chinese residents, (2) 15 years of age or older, and (3) Cantonese-speaking. For the landline phone calls, if multiple household members were eligible after successful contact, the one with the birthday closest to the interview date was selected. Further attempts were made by CATI to dial numbers for which there was “no answer”, “busy”, or had “eligible respondent not at home”. Oral informed consent was obtained at the beginning of each interview. All interviews were conducted during both working and non-working hours from 2 pm to 10 pm on weekdays and weekends. Response rates of 36.5% (cooperation rate = 77.2%, error = ±2.2% (95% CI)) and 33.8% (cooperation rate = 73.5%, error = ±3.1% (95% CI)) were recorded for survey 1 and survey 2, respectively. Detailed sampling information is documented in Supplemental Material A.

### Self-report measures

#### Disruptions to primary and secondary daily routines

The degree to which regular routines were disrupted during the last two weeks was assessed with one item on healthy eating and sleep (primary routines) and one item on socializing and leisure activities (secondary routines) in survey 1. Additional items on primary (household chores) and secondary (exercising/keeping active and work/study) routines were added in survey 2^12,19^. Respondents rated each item on an 11-point scale (0 = no disruptions, 10 = high level of disruptions).

#### Novel preventive routines

In survey 1, respondents reported whether or not, during the last two weeks, they had performed the following daily behaviors (no/yes): (1) worn a mask when they go out, (2) washed hands often, (3) avoided people with respiratory symptoms, (4) avoided going to crowded places, and (5) avoided using public transport. In survey 2, respondents were additionally asked to report whether they (6) stayed at home as much as possible, (7) used hand sanitizer, and (8) disinfected the house. These preventive behaviors were consistent with the recommendations made by the Centre for Health Protection^20^.

#### Anxiety

The 7-item Generalized Anxiety Disorder scale (GAD-7) was used to assess anxiety symptoms in the past 2 weeks^21^. Summed scores range from 0 to 21 (0 = not at all, 1 = on several days, 2 = on more than half of the days, 3 = nearly every day). Higher scores indicated greater severity of anxiety symptoms. The measure showed high internal consistency (Cronbach’s *α* = 0.92) and has been inversely correlated with self-rated physical and mental health in different populations^21^. Alphas were 0.93 in both administrations.

#### Depression

Depressive symptoms in the previous two weeks were assessed using the Chinese 9-item Patient Health Questionnaire (PHQ-9)^17,22^ on the same 4-point scale as the GAD-7. Higher scores indicated higher depressive symptoms (range = 0–27). Alphas were 0.86 and 0.85 in survey 1 and survey 2, respectively.

#### Demographics

We asked respondents’ age in years, gender, marital status (single, married/cohabitating, divorced/separated, widowed), education level, employment status, monthly household income, income change, and savings.

### Statistical analysis

Data was weighted by gender, age, and education level based on the Hong Kong population census 2019^23^. First, age and education level were recoded into groups to match the classifications in the census. Second, a weight for a specific group in a single demographic factor was calculated as the ratio of proportion of a specific group in the population to the proportion of the same group in the study sample. Third, a composite weight (i.e., the product of the three single weights) was assigned to each respondent. Missing data (<1%) was imputed by specifying a multivariate imputation model for each incomplete variable and generating imputations per variable iteratively^24^. Scores of 10 or higher on GAD-7^25^ were used to indicate clinical levels of anxiety symptoms. Scores of 10 or higher on PHQ-9 were used to indicate clinical levels of depressive symptoms^26^. The day-to-day prevalences of anxiety and depression were portrayed using nonparametric loess smoothing (locally weighted smoothing) to show the trends without assumption on the distributions and mapped onto the daily number of confirmed new cases during the study periods of the two surveys. Disruptions to daily routines was recoded into high (>1 SD of the mean), medium (within 1 SD of the mean), and low (<1 SD of the mean) in all analyses. Each novel preventive routine was a dichotomous variable (no = 0/yes = 1). The prevalences of anxiety and depression, disruptions to primary and secondary daily routines, and novel preventive routines were estimated with 95% confidence intervals (95% CI). Nonparametric Mann–Whitney *U* tests were used to examine differences in sociodemographics between respondents with and without anxiety and depression. Disruptions to daily routines and adoption of preventive routines, together with sociodemographics significant in the bivariate correlations were included in adjusted multivariable logistic regression models. Adjusted odds ratio (aOR) with 95% CI indicated the independent association of each correlate with each outcome. All analyses were performed using SPSS (Version 26; SPSS Inc., Chicago, IL). The significance level was set at *p* < 0.05 (two-tailed).

## Results

### Sample, prevalence, and trends

**Fig. 2:**
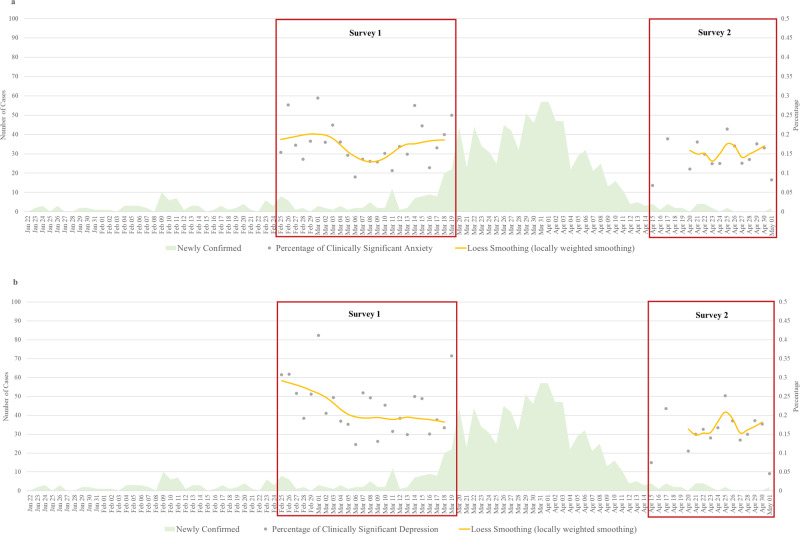
The trends of clinically significant anxiety and depression on the day-to-day number of confirmed new cases of the COVID-19. **a** = Anxiety. **b** = Depression. COVID-19 = Coronavirus disease 2019. Generalized Anxiety Disorder 7-item scale (GAD-7) scores at or exceeding 10 were used to define clinically significant anxiety. Patient Health Questionnaire-9 (PHQ-9) scores at or exceeding 10 were used to define clinically significant depression. Percentage of clinically significant anxiety/depression on a particular day represented the number of respondents with clinically significant anxiety/depression over the total number of respondents on that day. The loess smoothing depicts the trend of daily percentage of clinically significant anxiety/depression over the survey periods (survey 1: February 25–March 19, 2020; survey 2: April 15–May 1, 2020). The red frames indicate the trends of clinically significant anxiety/depression on the day-to-day number of confirmed new cases of the COVID-19. Light green area shows a trend of daily confirmed new cases of COVID-19 over the period of January 22–May 1, 2020. Data on daily confirmed new cases was obtained from the Centre for Health Protection website (https://www.chp.gov.hk/en/index.html).

The two samples resembled the population in terms of age group distribution, gender, education level, and other demographics (Supplemental Material B). The prevalence of anxiety (GAD-7) were 14.9% (95% CI = 13.8%–16.0%) and 14.0% (95% CI = 12.5%–15.5%), and the prevalence of depression (PHQ-9) were 19.6% (95% CI = 18.4–20.9%) and 15.3% (95% CI = 13.7%–16.9%). The prevalence of comorbid anxiety and depression were 10.6% (95% CI = 9.7%–11.6%) in survey 1 and 10.1% (95% CI = 8.8%–11.4%) in survey 2. The day-to-day rates of clinically significant anxiety showed a U-shape trend and depression showed a stable decreasing trend in survey 1, while both demonstrated an irregular pattern in survey 2 (Fig. 2). Sociodemographic characteristics consistently related to both anxiety and depression were age, marital status, income change, and savings, whereas education level, employment status, and monthly household income were associated with depression in both surveys (Supplemental Material B). 22%–24% experienced high disruptions to healthy eating and sleep and 28% experienced high disruptions to socializing and leisure activities, and there were similar percentages of disruptions to exercising/keeping active (22.8%) and work/study (20.0%) in survey 2 (Table 1). The most common preventive measures were wearing a mask when going out (>97%) and washing hand often (>92%), with notable increases in avoiding people with respiratory symptoms, avoiding going to crowded places, and avoiding using public transport from survey 1 to survey 2, and high rates of staying at home (90.6%), cleaning hands with disinfectants (88.7%), and disinfecting house (83.7%) in survey 2 (Table 1). Figure 1 illustrates major disease control policies and public data that reflect changes in population behavior.

**Table 1:** Prevalence of anxiety and depression, disruptions to primary and secondary daily routines, and novel preventive routines.

	Survey 1 Feb 25–Mar 19 (*n* = 4021)	Survey 2 Apr 15–May 1 (*n* = 2008)
Psychiatric symptoms^a^		
Anxiety^b^	600 (14.9%, 13.8%–16.0%)	281 (14.0%, 12.5%–15.5%)
Depression^c^	790 (19.6%, 18.4%–20.9%)	307 (15.3%, 13.7%–16.9%)
High disruptions to daily routines^d^		
Healthy eating and sleep	956 (23.8%, 22.5%–25.1%)	445 (22.2%, 20.3%–24.0%)
Socializing and leisure activities	1117 (27.8%, 26.4%–29.2%)	566 (28.2%, 26.2%–30.2%)
Healthy eating	NA	337 (16.8%, 15.2%–18.4%)
Sleep	NA	304 (15.1%, 13.6%–16.7%)
Household chores	NA	220 (11.0%, 9.6%–12.3%)
Leisure activities	NA	523 (26.1%, 24.1%–28.0%)
Exercising or keeping active	NA	458 (22.8%, 21.0%–24.7%)
Socializing	NA	480 (23.9%, 22.0%–25.8%)
Work or study	NA	402 (20.0%, 18.3%–21.8%)
Adoption of preventive routines^e^		
Wear a mask when I go out	3924 (97.6%, 97.1%–98.1%)	1992 (99.2%, 98.8%–99.6%)
Wash hands often	3719 (92.5%, 91.7%–93.3%)	1928 (96.0%, 95.2%–96.9%)
Avoid people with respiratory symptoms	2799 (69.6%, 68.2%–71.0%)	1761 (87.7%, 86.3%–89.1%)
Avoid going to crowded places	3232 (80.4%, 79.2%–81.6%)	1754 (87.3%, 85.9%–88.8%)
Avoid using public transport	2159 (53.7%, 52.1%–55.2%)	1242 (61.9%, 59.7%–64.0%)
Stay at home as much as possible	NA	1819 (90.6%, 89.3%–91.9%)
Use hand sanitizer	NA	1781 (88.7%, 87.3%–90.1%)
Disinfect house	NA	1680 (83.7%, 82.0%–85.3%)

Data are *n* (%, 95% confidence interval). Prevalence was weighted by gender, age, and education level based on the Hong Kong population census 2019. All dates are in 2020.

*NA* not applicable (question was not asked in the survey).

^a^Numbers and prevalence represent respondents that had clinical level of psychiatric symptoms.

^b^The 7-item Generalized Anxiety Disorder scale (GAD-7) scores at or exceeding 10 were used to define clinical levels of anxiety symptoms.

^c^The 9-item Patient Health Questionnaire (PHQ-9) scores at or exceeding 10 were used to define clinical levels of depressive symptoms.

^d^Numbers and proportions represent respondents that were highly disrupted to daily routines.

^e^Numbers and proportions represent respondents that adopted the preventive routines.

### Multivariable analyses

#### Survey 1

Anxiety was significantly positively correlated with high/medium disruptions to healthy eating, sleep, socializing, and leisure activities (compared with low disruptions), avoiding people with respiratory symptoms, female gender, and income decline (Table 2). Significant positive correlates of depression were high/medium disruptions to healthy eating, sleep, socializing and leisure activities (compared with low disruptions), not avoiding going to crowded places, age 25 years or older (compared with age 15–24 years), female gender, being unemployed, and income decline (Table 2).

**Table 2:** Multivariable logistic regression examining correlates of anxiety and depression.

	Odds Ratio (95% Confidence Interval)							
	Survey 1, Feb 25–Mar 19 (*n* = 4021)				Survey 2, Apr 15–May 1 (*n* = 2008)			
	Anxiety^a^	*p*	Depression^b^	*p*	Anxiety^a^	*p*	Depression^b^	*p*
Gender^c^								
Male	1.00		1.00		–		–	
Female	1.31 (1.08–1.59)	0.006	1.35 (1.13–1.62)	0.001	–		–	
Age								
15–24	1.00		1.00		1.00		1.00	
25–34	1.29 (0.93–1.80)	0.131	1.59 (1.12–2.27)	0.010	0.99 (0.56–1.74)	0.970	0.87 (0.49–1.54)	0.631
35–44	1.17 (0.82–1.69)	0.387	1.55 (1.06–2.25)	0.023	1.03 (0.55–1.92)	0.922	1.21 (0.66–2.23)	0.535
45–64	0.79 (0.56–1.13)	0.194	1.40 (0.99–2.00)	0.059	0.74 (0.41–1.32)	0.306	0.90 (0.50–1.61)	0.715
65 or above	0.92 (0.60–1.39)	0.679	2.03 (1.38–3.00)	<0.001	0.60 (0.32–1.15)	0.124	0.84 (0.43–1.62)	0.601
Marital status								
Married	1.00		1.00		1.00		1.00	
Unmarried/divorced/widowed	1.12 (0.90–1.39)	0.335	1.14 (0.93–1.40)	0.194	1.33 (0.95–1.87)	0.101	1.29 (0.93–1.79)	0.121
Education level^c^								
Tertiary or above	1.00		1.00		–		1.00	
Secondary	0.89 (0.71–1.11)	0.295	1.19 (0.95–1.48)	0.129	–		1.47 (1.01–2.15)	0.045
Primary or below	0.83 (0.56–1.23)	0.351	0.89 (0.63–1.27)	0.526	–		1.99 (1.10–3.61)	0.023
Employment^c^								
Employed	–		1.00		1.00		1.00	
Dependent	–		1.07 (0.84–1.35)	0.582	0.85 (0.57–1.28)	0.449	0.85 (0.58–1.26)	0.428
Unemployed	–		1.52 (1.05–2.20)	0.026	2.15 (1.21–3.79)	0.009	1.97 (1.12–3.44)	0.018
Monthly household income (HK$)^c^								
$80,000 or above	–		1.00		1.00		1.00	
$60,000–$79,999	–		0.88 (0.56–1.36)	0.555	1.09 (0.50–2.36)	0.836	1.11 (0.50–2.48)	0.792
$40,000–$59,999	–		1.00 (0.71–1.41)	0.991	1.52 (0.82–2.81)	0.180	1.64 (0.87–3.08)	0.126
$20,000–$39,999	–		0.93 (0.66–1.30)	0.659	1.04 (0.58–1.86)	0.907	1.47 (0.81–2.68)	0.207
$19,999 or below	–		0.89 (0.63–1.28)	0.537	1.09 (0.59–2.01)	0.793	1.29 (0.69–2.41)	0.422
Income change								
Stable/Increase	1.00		1.00		1.00		1.00	
Decrease	1.45 (1.19–1.77)	<0.001	1.48 (1.23–1.79)	<0.001	1.28 (0.95–1.73)	0.107	1.00 (0.75–1.34)	0.996
Savings (HK$)								
$3,000,000 or above	1.00		1.00		1.00		1.00	
$2,000,000–$2,999,999	0.76 (0.35–1.64)	0.486	1.06 (0.52–2.15)	0.882	0.50 (0.11–2.32)	0.379	0.46 (0.10–2.10)	0.316
$1,000,000–$1,999,999	0.64 (0.37–1.11)	0.114	0.68 (0.40–1.17)	0.164	0.57 (0.18–1.87)	0.357	0.69 (0.22–2.10)	0.510
$500,000–$999,999	0.62 (0.37–1.03)	0.063	0.85 (0.52–1.39)	0.519	1.78 (0.69–4.61)	0.235	1.56 (0.62–3.96)	0.347
$200,000–$499,999	0.71 (0.45–1.13)	0.146	1.00 (0.64–1.57)	0.985	1.37 (0.56–3.31)	0.488	1.60 (0.68–3.76)	0.280
Less than $200,000	0.92 (0.60–1.40)	0.689	1.42 (0.93–2.18)	0.103	1.92 (0.84–4.39)	0.121	1.95 (0.87–4.34)	0.103
None	0.89 (0.57–1.39)	0.609	1.20 (0.77–1.87)	0.429	2.61 (1.11–6.14)	0.028	2.65 (1.15–6.10)	0.022
Disruptions to healthy eating and sleep								
Low	1.00		1.00		1.00		1.00	
Medium	2.26 (1.64–3.10)	<0.001	2.45 (1.86–3.24)	<0.001	2.23 (1.16–4.30)	0.016	2.27 (1.26–4.06)	0.006
High	5.03 (3.62–6.99)	<0.001	7.51 (5.60–10.07)	<0.001	6.67 (3.33–13.38)	<0.001	6.29 (3.35–11.79)	<0.001
Disruptions to socializing and leisure activities								
Low	1.00		1.00		1.00		1.00	
Medium	1.73 (1.24–2.42)	0.001	1.82 (1.37–2.42)	<0.001	2.04 (1.01–4.10)	0.046	1.71 (0.95–3.08)	0.074
High	4.18 (2.97–5.89)	<0.001	3.09 (2.30–4.16)	<0.001	5.12 (2.49–10.54)	<0.001	3.12 (1.68–5.77)	<0.001
Disruptions to household chores								
Low	NA		NA		1.00		1.00	
Medium	NA		NA		0.65 (0.43–0.99)	0.043	1.03 (0.69–1.54)	0.878
High	NA		NA		1.12 (0.66–1.90)	0.670	1.37 (0.82–2.29)	0.234
Disruptions to exercising or keeping active								
Low	NA		NA		1.00		1.00	
Medium	NA		NA		0.77 (0.49–1.22)	0.268	0.70 (0.46–1.06)	0.089
High	NA		NA		1.36 (0.83–2.22)	0.222	1.41 (0.89–2.23)	0.138
Disruptions to work or study								
Low	NA		NA		1.00		1.00	
Medium	NA		NA		1.36 (0.86–2.14)	0.190	0.89 (0.59–1.35)	0.575
High	NA		NA		2.15 (1.28–3.59)	0.004	1.77 (1.10–2.84)	0.019
Adoption of all preventive measures								
Yes	1.00		1.00		1.00		1.00	
No	1.02 (0.68–1.53)	0.927	0.89 (0.62–1.27)	0.506	1.12 (0.67–1.87)	0.653	0.92 (0.56–1.50)	0.727
Wear a mask when I go out								
Yes	1.00		1.00		1.00		1.00	
No	1.50 (0.79–2.86)	0.212	1.65 (0.97–2.82)	0.067	2.09 (0.42–10.51)	0.370	0.99 (0.20–4.92)	0.992
Wash hands often								
Yes	1.00		1.00		1.00		1.00	
No	0.98 (0.65–1.49)	0.931	1.39 (0.99–1.96)	0.056	0.87 (0.41–1.84)	0.715	0.69 (0.33–1.43)	0.314
Avoid people with respiratory symptoms								
Yes	1.00		1.00		1.00		1.00	
No	0.71 (0.53–0.95)	0.020	1.09 (0.85–1.41)	0.483	0.84 (0.49–1.43)	0.518	0.94 (0.57–1.55)	0.812
Avoid going to crowded places								
Yes	1.00		1.00		1.00		1.00	
No	0.84 (0.64–1.12)	0.246	1.29 (1.01–1.65)	0.038	1.04 (0.64–1.69)	0.890	0.78 (0.48–1.26)	0.314
Avoid using public transport								
Yes	1.00		1.00		1.00		1.00	
No	1.02 (0.71–1.48)	0.895	1.06 (0.78–1.45)	0.720	0.82 (0.52–1.29)	0.388	0.83 (0.54–1.29)	0.414
Stay at home as much as possible								
Yes	NA		NA		1.00		1.00	
No	NA		NA		1.28 (0.74–2.23)	0.379	1.51 (0.90–2.53)	0.119
Use hand sanitizer								
Yes	NA		NA		1.00		1.00	
No	NA		NA		1.04 (0.60–1.77)	0.900	1.06 (0.64–1.75)	0.819
Disinfect house								
Yes	NA		NA		1.00		1.00	
No	NA		NA		0.58 (0.36–0.93)	0.023	0.87 (0.56–1.34)	0.523

Data was weighted by gender, age, and education level based on the Hong Kong population census 2019. All dates are in 2020.

*NA* not applicable (question was not asked in the survey).

^a^The 7-item Generalized Anxiety Disorder scale (GAD-7) scores at or exceeding 10 were used to define clinical levels of anxiety symptoms.

^b^The 9-item Patient Health Questionnaire (PHQ-9) scores at or exceeding 10 were used to define clinical levels of depressive symptoms.

^c^Demographic variables that were not significant in bivariable analyses (Supplemental Material B) were not included in the multivariable logistic regression.

#### Survey 2

Anxiety was significantly positively associated with high and/or medium disruptions to healthy eating, sleep, socializing, leisure activities, and work/study (compared with low disruptions), disinfecting the house, being unemployed, and a lack of savings (Table 2). Significant positive correlates of depression were high and/or medium disruptions to healthy eating, sleep, socializing, leisure activities, and work/study (compared with low disruptions), primary and secondary education, being unemployed, and a lack of savings (Table 2).

## Discussion

We report a large population-representative study of the mental impact of COVID-19 in a region (*n* ≥ 6000), addressing the urgent need to collect high-quality data on the mental health toll of this pandemic, over time^27^. We assessed anxiety and depressive symptoms using validated instruments with established population norms. 14.9% of local Hong Kong residents experienced significant anxiety symptoms and 19.6% experienced significant depressive symptoms at the beginning of the pandemic (survey 1: February 25–March 19); 14% reporting anxiety and 15.3% reporting depression following a period of high incidence (survey 2: April 15–May 1). Disruptions to different daily routines was a significant correlate of increased odds of anxiety and depression across survey 1 and 2. Absence of new preventive routines was associated with decreased odds of anxiety in both surveys and increased odds of depression in survey 1 only. In both surveys, lower socioeconomic status including lower education level, being unemployed, and a lack of savings was associated with increased odds of anxiety and/or depression.

Hong Kong faced a public mental health crisis during and after the SARS epidemic (i.e., May–June, 2003)^18^. The current prevalences of anxiety and depression during the COVID-19 were higher than during the SARS and other major societal challenges including large-scale civil unrests in 2014^26^. The prevalence of depression was at least four times higher than during periods without significant social, political, and public health crises^17^. The effort in containing the virus and preventing community outbreak in Hong Kong has been largely seen as satisfactory, with 1094 confirmed cases and four death cases at the time of writing (June 3, 2020). Higher prevalences of depression and anxiety were observed in survey 1 possibly because there were a lot of uncertainties towards COVID-19 and very limited infection control measures were implemented at that time. During survey 2, control measures were implemented intensively and the number of confirmed new cases remained stably low. It is possible that lower symptoms of depression and anxiety at survey 2 relative to survey 1 were due to reduced stress and distress surrounding COVID-19. Because the incidence and mortality rates in Hong Kong have been relatively low and stable since the outbreak in January, 2020, we might expect higher prevalence of mental health problems in regions that are more severely affected by the pandemic socially and economically. There is evidence showing that psychological distress was higher whereas perceived health and well-being were lower during and after home confinement in China, the country most affected by the early phase of the outbreak^28^.

The current findings extend previous evidence on the importance of sustaining daily routines for mental health from natural disasters^9,10^ and humanitarian crises^11,19^ to the current pandemic^29^. We used a previously validated self-report instrument^12^ and found that disruptions to regularity of primary routines (healthy eating and sleep) and secondary routines (socializing and leisure activities) was associated with higher odds of both anxiety and depression in both survey 1 and survey 2. The daily routines most relevant to mental health are healthy eating, sleep, socializing, leisure activities, and work/study. We suggest that the extent to which these important daily routines are disrupted is closely linked to higher mental health problems throughout different phases of a pandemic. Our findings provide initial evidence for international recommendations on the importance of regular daily routines for population mental health during the COVID-19^30^. Work on infectious diseases finds that the level of perceived risk is a further additional risk for mental well-being during a pandemic^31^. To test this we re-ran our analyses and found that the associations between disrupted daily routines and anxiety/depression remained significant after controlling for perceived infection risk and life/health and economic threat associated with the COVID-19, suggesting regularizing daily routines could benefit mental health across those experiencing different levels of stress (Supplemental Material C).

In our studies, the addition of new non-pharmaceutical preventive routines was important for psychological adaptation in the acute phase of a pandemic. Respondents who did not avoid going to crowded places were at increased odds of depression in survey 1 but not survey 2. One explanation for the findings in survey 1 is that people who are depressed are more likely to be fatalistic about preventing infection or less efficacious in adopting preventive measures^2^. Another explanation is that these officially recommended and enforced preventive routines are seen as effective in reducing the risk of infection and thus confer better mental health through restoring a sense of normalcy, meaning, and engagement in everyday life^32,33^. Persons who do not adopt those routines might not benefit from their potential mental health benefit and thus report higher levels of depression. It is possible that the preventive routines, novel during survey 1, had become part of the daily routines for most people in survey 2, and therefore their positive mental health impact needs to be captured in term of regularity^11,12^ not merely their presence or absence as the pandemic unfolds.

Our findings, nevertheless, also show that the adoption of specific preventive routines, namely avoiding people with respiratory symptoms (survey 1) and disinfecting the house (survey 2) did not reduce and can even increase anxiety. This suggests that there is a need to consider the links of anxiety and depression to preventive routines separately. Risk-avoidant behaviors are common adopted for reducing negative emotions among persons with anxiety, but without a safe, effective, and affordable vaccine in sight, a heavy responsibility is placed upon people to adopt preventive measures for self-protection and the prevention of a community outbreak, and the demands and changes in behaviors could be excessive and contribute to anxious feelings^34^. It is also important to note that the psychological meaning of preventive measures, for example the regular use of face masks, will vary across sociocultural contexts with different levels of prior exposure, warranting cross-cultural comparisons^13,35^.

The current study found higher odds of anxiety and depression among females relative to males in survey 1, while no gender difference was found on the prevalence of depression in a population-based study conducted in 2019 in Hong Kong^16^. Gender differences might exist in biological stress responses and coping strategies in the acute phase of COVID-19^36,37^. Respondents younger than 25 years of age showed lower odds of depression compared with those aged 25 years or older in survey 1 but not in survey 2, possible because there was a belief that young people are less vulnerable to COVID-19 infection^38^. In survey 2, a period with intensive infection control measures and stable and low incidence rates, there was no age difference in the odds of depression.

We documented a socioeconomic gradient whereby lower education levels, being unemployed, and lower economic status were consistently associated with greater odds of anxiety and depression. Amid pandemic outbreaks, mental health varies as a function of socioeconomic status (SES), and stress amongst people with lower SES could be compounded by other chronic or societal stressors in their daily life^39^. Many societies face the shortage and soaring prices of protective equipment, making this less affordable and accessible for low-income families. Special work arrangements to avoid the risk of infection, such as working from home, are not applicable to many low-wage workers in manual labor or unskilled occupations, who are thus exposed to higher risk of infection as well as poor mental health. These workers are also more susceptible to the potential layoffs, company closures, and delayed wages triggered by the pandemic and evidenced in both high-income countries (HICs) and low- and middle-income countries (LMICs)^3,27^. Home confinement increases family time, but it can stress already tight household budgets and increase the risk of domestic violence in low-income families^3^. School closure during the pandemic can create further problems amongst poorer families, especially where children have relatively limited access to compensational educational resources such as equipment for online learning, and where parents might be reluctant to give up workdays for childcare^40^. Attention is needed to limit the burden of mental illness amongst already disadvantaged groups in society.

The current study collected two population representative samples during two different phases of the COVID-19 pandemic in a densely populated urban center. Some limitations however should be noted when interpreting our findings. First, screening rather than clinician diagnoses was used to assess psychiatric symptoms in this study. The instruments we used for assessing anxiety and depression were well-validated and widely used with established norms in Greater China^17,22,26,28^. However it is practically impossible to conduct face-to-face interviews amongst representative samples of a population during a pandemic. Second, we only assessed healthy eating, sleep, socializing, and leisure activities as the key daily routines disrupted by quarantine and physical distancing in survey 1. Other routines such as household chores, exercising, and work/study involvement^6^ were not included. Third, a more comprehensive list of common non-pharmaceutical preventive measures^41^ were assessed in survey 2 only. Fourth, no information was provided on respondents’ prior or current quarantine status, which could be at home or temporary assigned places including designated hotels, holiday camps, or newly constructed vacant public estates. Fifth, the simultaneous social unrest could compound the positive associations between COVID-19 stress and anxiety and depression^42^. Hong Kong has been undergoing widespread social unrest since June, 2019. We found that the prevalence of depression was actually higher than those that have been documented during previous and the current social unrest in Hong Kong^26,43^. Last but not least, there is evidence showing that heavy social media use was one of the risk factors of probable depression during social unrest in Hong Kong^43^. Future study should consider the potential impact of social media on population mental health during COVID-19 in Hong Kong.

Notwithstanding these limitations, this study offers some of the first supportive evidence for the recommendation of regularizing daily routines to assist mental health during the COVID-19. Our population-representative data were obtained in one of the world’s most densely populated cities facing a heightened challenge from the pandemic. Extrapolating the estimates on anxiety and depression based on data of survey 1 and survey 2 to a population of 7.5 million people at least 1.05 million Hong Kong people could have anxiety and 1.15 million could have depression. In regions more severely affected by the pandemic, prompt government responses to disseminate guidelines on regularizing daily routines are urgently needed. This could be realistically strengthened face-to-face by a national team of trained community health workers or nationwide voluntary schemes such as GoodSam in the UK, along with regular community health programs targeted at disadvantaged groups such as older adults and essential low-wage workers at increased risk of infection. These cost-effective interventions could inoculate the populations against heightened risk of poor mental health, reducing in turn the burden on already stressed medical care sectors during and after the pandemic.

## Supplementary information

Supplemental Materials
