# Supplementary material for: Psychiatric symptoms and behavioral adjustment during the COVID-19 pandemic: evidence from two population-representative cohorts
Source: Transl Psychiatry. 2021 Mar 17;11:174. doi: 10.1038/s41398-021-01279-w (PMC7967107; doi:10.1038/s41398-021-01279-w)
Supplement: Supplementary file 1 — Supplemental Materials [file 41398_2021_1279_MOESM1_ESM.docx]

Supplemental Material A. Details of the interviews information.

In survey 1 (February 25–March 19, 2020), a total of 92 509 telephone numbers were attempted, 38 538 (41.7%) of them were ineligible for interview (i.e., invalid, non-resident/business telephone, fax numbers, no eligible respondent) and 48 765 (52.7%) were unconﬁrmed eligible. Among the 5 206 (5.6%) eligible numbers, interviews were completed for 4 021 (77.2%); 884 (17.0%) refused and 301 (5.8%) eligible respondents did not complete the interviews.

In survey 2 (April 15–May 1, 2020), a total of 59 650 telephone numbers were attempted, 24 706 (41.4%) of them were ineligible for interview (i.e., invalid, non-resident/business telephone, fax numbers, no eligible respondent) and 32 213 (54.0%) were unconﬁrmed eligible. Among the 2 731 (4.6%) eligible numbers, interviews were completed for 2 008 (73.5%); 416 (15.2%) refused and 307 (11.2%) eligible respondents did not complete the interviews.

Response rate = Completed / [Eligibles + Unknown x Eligibles / (Eligibles + Ineligibles)]

Cooperation rate = Completed / Eligibles

Supplemental Material B

Demographics and bivariable analyses.

|  | **Survey 1**  **Feb 25–Mar 19** | | | | | **Survey 2**  **Apr 15–May 1** | | | | | |
| --- | --- | --- | --- | --- | --- | --- | --- | --- | --- | --- | --- |
| **Variable** |  | **Anxiety†** | | **Depressionǂ** | |  | **Anxiety†** | | **Depressionǂ** | |  |
|  | ***N*** | **%** | ***p*** | **%** | ***p*** | ***N*** | **%** | ***p*** | **%** | ***p*** |  |
| **Total** | 4 021 | 14.9 |  | 19.6 |  | 2 008 | 14.0 |  | 15.3 |  |  |
| **Gender** |  |  | <0.001 |  | <0.001 |  |  | 0.347 |  | 0.051 |  |
| Male | 1 902 | 12.9 |  | 17.2 |  | 950 | 13.8 |  | 14.1 |  |  |
| Female | 2 119 | 16.7 |  | 21.8 |  | 1 058 | 14.1 |  | 16.4 |  |  |
| **Age** |  |  | <0.001 |  | <0.001 |  |  | <0.001 |  | 0.005 |  |
| 15–24 | 469 | 20.7 |  | 21.6 |  | 216 | 19.8 |  | 19.1 |  |  |
| 25–34 | 599 | 21.5 |  | 23.3 |  | 337 | 18.7 |  | 15.9 |  |  |
| 35–44 | 681 | 17.7 |  | 20.5 |  | 335 | 16.3 |  | 17.7 |  |  |
| 45–64 | 1 443 | 11.5 |  | 17.5 |  | 704 | 11.7 |  | 13.5 |  |  |
| 65 or above | 829 | 10.6 |  | 18.9 |  | 416 | 9.2 |  | 13.8 |  |  |
| **Marital status** |  |  | <0.001 |  | <0.001 |  |  | <0.001 |  | 0.001 |  |
| Married | 2 381 | 12.6 |  | 17.4 |  | 1 199 | 11.4 |  | 13.1 |  |  |
| Unmarried/divorced/widowed | 1 640 | 18.3 |  | 22.9 |  | 809 | 17.8 |  | 18.6 |  |  |
| **Education level** |  |  | 0.004 |  | 0.028 |  |  | 0.094 |  | <0.001 |  |
| Tertiary or above | 1 379 | 17.2 |  | 17.5 |  | 689 | 12.0 |  | 10.9 |  |  |
| Secondary | 2 087 | 14.3 |  | 21.2 |  | 1 055 | 15.7 |  | 17.1 |  |  |
| Primary or below | 555 | 11.6 |  | 19.2 |  | 264 | 12.1 |  | 19.5 |  |  |
| **Employment** |  |  | 0.575 |  | 0.009 |  |  | 0.033 |  | <0.001 |  |
| Employed | 2 187 | 14.8 |  | 18.2 |  | 1 135 | 12.8 |  | 13.3 |  |  |
| Dependent | 1 633 | 14.3 |  | 20.1 |  | 765 | 12.8 |  | 15.6 |  |  |
| Unemployed | 201 | 21.5 |  | 32.3 |  | 108 | 34.4 |  | 34.3 |  |  |
| **Monthly household income (HK$)** |  |  | 0.374 |  | <0.001 |  |  | 0.001 |  | <0.001 |  |
| $80 000 or above | 474 | 14.8 |  | 15.2 |  | 264 | 7.4 |  | 6.6 |  |  |
| $60 000–$79 999 | 302 | 14.2 |  | 14.1 |  | 139 | 11.9 |  | 10.5 |  |  |
| $40 000–$59 999 | 751 | 17.3 |  | 19.8 |  | 354 | 14.4 |  | 13.4 |  |  |
| $20 000–$39 999 | 1 240 | 14.5 |  | 20.1 |  | 604 | 14.3 |  | 16.9 |  |  |
| $19 999 or below | 1 254 | 14.1 |  | 22.1 |  | 647 | 16.5 |  | 19.4 |  |  |
| **Income change** |  |  | <0.001 |  | <0.001 |  |  | <0.001 |  | <0.001 |  |
| Stable/Increase | 2 833 | 12.8 |  | 16.9 |  | 1 160 | 10.8 |  | 13.0 |  |  |
| Decrease | 1 188 | 20.1 |  | 26.3 |  | 848 | 18.3 |  | 18.4 |  |  |
| **Savings (HK$)** |  |  | <0.001 |  | <0.001 |  |  | <0.001 |  | <0.001 |  |
| $3 000 000 or above | 283 | 12.4 |  | 13.2 |  | 159 | 5.5 |  | 5.6 |  |  |
| $2 000 000–$2 999 999 | 99 | 11.1 |  | 14.1 |  | 60 | 4.3 |  | 4.3 |  |  |
| $1 000 000–$1 999 999 | 316 | 10.1 |  | 10.8 |  | 151 | 3.8 |  | 4.4 |  |  |
| $500 000–$999 999 | 385 | 12.0 |  | 14.8 |  | 170 | 10.5 |  | 9.8 |  |  |
| $200 000–$499 999 | 634 | 14.1 |  | 17.8 |  | 266 | 12.0 |  | 13.3 |  |  |
| Less than $200 000 | 1 468 | 17.8 |  | 24.3 |  | 807 | 15.9 |  | 17.3 |  |  |
| None | 836 | 14.9 |  | 21.1 |  | 395 | 21.6 |  | 24.6 |  |  |

Data was weighted by gender, age, and education level based on the Hong Kong population census 2019. All dates are in 2020. †The 7-item Generalized Anxiety Disorder scale (GAD-7) scores at or exceeding 10 were used to define clinical levels of anxiety symptoms. **ǂ**The 9-item Patient Health Questionnaire (PHQ-9) scores at or exceeding 10 were used to define clinical levels of depressive symptoms.

Supplemental Material C

Prevalence of anxiety and depression, disruptions to primary and secondary daily routines, novel preventive routines, and COVID-19-related perceptions.

|  | **Survey 1**  **Feb 25–Mar 19**  **(n=4 021)** | **Survey 2**  **Apr 15–May 1**  **(n=2 008)** |
| --- | --- | --- |
| **Psychiatric symptoms*** |  |  |
| Anxiety† | 600 (14.9%, 13.8%–16.0%) | 281 (14.0%, 12.5%–15.5%) |
| Depression**ǂ** | 790 (19.6%, 18.4%–20.9%) | 307 (15.3%, 13.7%–16.9%) |
| **High disruptions to daily routines§** |  |  |
| Healthy eating and sleep | 956 (23.8%, 22.5%–25.1%) | 445 (22.2%, 20.3%–24.0%) |
| Socializing and leisure activities | 1 117 (27.8%, 26.4%–29.2%) | 566 (28.2%, 26.2%–30.2%) |
| Healthy eating | NA | 337 (16.8%, 15.2%–18.4%) |
| Sleeping | NA | 304 (15.1%, 13.6%–16.7%) |
| Household chores | NA | 220 (11.0%, 9.6%–12.3%) |
| Leisure activities | NA | 523 (26.1%, 24.1%–28.0%) |
| Exercising or keeping active | NA | 458 (22.8%, 21.0%–24.7%) |
| Socializing | NA | 480 (23.9%, 22.0%–25.8%) |
| Work or study | NA | 402 (20.0%, 18.3%–21.8%) |
| **Adoption of preventive routines**¶ |  |  |
| Wear a mask when I go out | 3 924 (97.6%, 97.1%–98.1%) | 1 992 (99.2%, 98.8%–99.6%) |
| Wash hands often | 3 719 (92.5%, 91.7%–93.3%) | 1 928 (96.0%, 95.2%–96.9%) |
| Avoid people with respiratory symptoms | 2 799 (69.6%, 68.2%–71.0%) | 1 761 (87.7%, 86.3%–89.1%) |
| Avoid going to crowded places | 3 232 (80.4%, 79.2%–81.6%) | 1 754 (87.3%, 85.9%–88.8%) |
| Avoid using public transport | 2 159 (53.7%, 52.1%–55.2%) | 1 242 (61.9%, 59.7%–64.0%) |
| Stay at home as much as possible | NA | 1 819 (90.6%, 89.3%–91.9%) |
| Use hand sanitizer | NA | 1 781 (88.7%, 87.3%–90.1%) |
| Disinfect house | NA | 1 680 (83.7%, 82.0%–85.3%) |
| **COVID-19 related perceptions** |  |  |
| Worry about infecting the COVID-19****** | 3 548 (88.2%, 87.2%–89.2%) | NA |
| Threat of financial strain*** | NA | 1 207 (60.1%, 58.0%–62.2%) |
| Reduced income*** | NA | 1 310 (65.3%, 63.2%–67.3%) |
| Reduced savings*** | NA | 1 407 (70.1%, 68.1%–72.1%) |
| Threat of life*** | NA | 1 722 (85.8%, 84.3%–87.3%) |
| Long-term health problems*** | NA | 1 836 (91.4%, 90.2%–92.6%) |
| Side-effects of treatment*** | NA | 1 800 (89.6%, 88.3%–91.0%) |

Data are n (%, 95% confidence interval). Prevalence was weighted by gender, age, and education level based on the Hong Kong population census 2019. All dates are in 2020. NA=not applicable (question was not asked in the survey). COVID-19=coronavirus disease 2019. *Numbers and prevalence represent respondents that had clinical level of psychiatric symptoms. †The 7-item Generalized Anxiety Disorder scale (GAD-7) scores at or exceeding 10 were used to define clinical levels of anxiety symptoms. ǂThe 9-item Patient Health Questionnaire (PHQ-9) scores at or exceeding 10 were used to define clinical levels of depressive symptoms. §Numbers and proportions represent respondents that were highly disrupted to daily routines. ¶Numbers and proportions represent respondents that adopted the preventive routines. **Number and proportion represent respondents that answered somewhat, quite a bit, and very much worry about infecting the COVID-19. ***Numbers and proportions represented respondents that agreed and strongly agreed the COVID-19 related perceptions.

Multivariable logistic regression examining correlates of anxiety and depression with COVID-19-related perceptions.

|  | **Odds Ratio (95% Confidence Interval)** | | | | | | | |
| --- | --- | --- | --- | --- | --- | --- | --- | --- |
|  | **Survey 1, Feb 25–Mar 19**  **(n=4 021)** | | | | **Survey 2, Apr 15–May 1**  **(n=2 008)** | | | |
|  | **Anxietyǂ** | ***P*** | **Depression**§ | ***P*** | **Anxietyǂ** | ***P*** | **Depression**§ | ***P*** |
| **Gender**¶ |  |  |  |  |  |  |  |  |
| Male | 1.00 |  | 1.00 |  | .. |  | .. |  |
| Female | 1.29 (1.06–1.57) | 0.010 | 1.34 (1.12–1.61) | 0.002 | .. |  | .. |  |
| **Age** |  |  |  |  |  |  |  |  |
| 15–24 | 1.00 |  | 1.00 |  | 1.00 |  | 1.00 |  |
| 25–34 | 1.31 (0.94–1.83) | 0.113 | 1.61 (1.13–2.29) | 0.009 | 0.99 (0.56–1.74) | 0.959 | 0.87 (0.49–1.53) | 0.621 |
| 35–44 | 1.21 (0.84–1.74) | 0.308 | 1.56 (1.07–2.28) | 0.020 | 1.07 (0.57–2.01) | 0.830 | 1.28 (0.69–2.36) | 0.433 |
| 45–64 | 0.84 (0.59–1.20) | 0.336 | 1.43 (1.01–2.04) | 0.046 | 0.75 (0.41–1.35) | 0.337 | 0.92 (0.51–1.67) | 0.793 |
| 65 or above | 0.96 (0.64–1.46) | 0.861 | 2.07 (1.41–3.05) | <0.001 | 0.68 (0.35–1.30) | 0.244 | 0.95 (0.49–1.85) | 0.882 |
| **Marital status** |  |  |  |  |  |  |  |  |
| Married | 1.00 |  | 1.00 |  | 1.00 |  | 1.00 |  |
| Unmarried/divorced/widowed | 1.15 (0.92–1.43) | 0.230 | 1.15 (0.94–1.41) | 0.165 | 1.44 (1.02–2.04) | 0.040 | 1.39 (1.00–1.93) | 0.053 |
| **Education level**¶ |  |  |  |  |  |  |  |  |
| Tertiary or above | 1.00 |  | 1.00 |  | .. |  | 1.00 |  |
| Secondary | 0.89 (0.71–1.12) | 0.311 | 1.19 (0.95–1.48) | 0.128 | .. |  | 1.46 (1.00–2.15) | 0.053 |
| Primary or below | 0.84 (0.56–1.25) | 0.382 | 0.90 (0.63–1.28) | 0.544 | .. |  | 1.98 (1.08–3.62) | 0.027 |
| **Employment**¶ |  |  |  |  |  |  |  |  |
| Employed | .. |  | 1.00 |  | 1.00 |  | 1.00 |  |
| Dependent | .. |  | 1.07 (0.85–1.36) | 0.560 | 0.88 (0.58–1.33) | 0.552 | 0.88 (0.59–1.31) | 0.534 |
| Unemployed | .. |  | 1.54 (1.06–2.22) | 0.023 | 2.04 (1.14–3.65) | 0.016 | 1.85 (1.05–3.26) | 0.034 |
| **Monthly household income (HK$)**¶ |  |  |  |  |  |  |  |  |
| $80 000 or above | .. |  | 1.00 |  | 1.00 |  | 1.00 |  |
| $60 000–$79 999 | .. |  | 0.87 (0.56–1.35) | 0.537 | 1.14 (0.52–2.51) | 0.743 | 1.17 (0.52–2.63) | 0.696 |
| $40 000–$59 999 | .. |  | 1.00 (0.71–1.41) | 0.994 | 1.39 (0.75–2.58) | 0.302 | 1.48 (0.78–2.79) | 0.232 |
| $20 000–$39 999 | .. |  | 0.93 (0.66–1.30) | 0.653 | 0.89 (0.49–1.62) | 0.698 | 1.28 (0.70–2.36) | 0.421 |
| $19 999 or below | .. |  | 0.90 (0.63–1.28) | 0.547 | 0.86 (0.46–1.60) | 0.625 | 1.02 (0.54–1.93) | 0.947 |
| **Income change** |  |  |  |  |  |  |  |  |
| Stable/Increase | 1.00 |  | 1.00 |  | 1.00 |  | 1.00 |  |
| Decrease | 1.45 (1.18–1.77) | <0.001 | 1.48 (1.23–1.78) | <0.001 | 0.98 (0.71–1.37) | 0.911 | 0.77 (0.56–1.06) | 0.110 |
| **Savings (HK$)** |  |  |  |  |  |  |  |  |
| $3 000 000 or above | 1.00 |  | 1.00 |  | 1.00 |  | 1.00 |  |
| $2 000 000–$2 999 999 | 0.75 (0.35–1.61) | 0.459 | 1.06 (0.52–2.15) | 0.877 | 0.51 (0.11–2.40) | 0.398 | 0.46 (0.10–2.12) | 0.319 |
| $1 000 000–$1 999 999 | 0.65 (0.38–1.12) | 0.122 | 0.69 (0.40–1.17) | 0.170 | 0.62 (0.19–2.06) | 0.435 | 0.74 (0.24–2.29) | 0.603 |
| $500 000–$999 999 | 0.61 (0.36–1.01) | 0.055 | 0.85 (0.52–1.39) | 0.518 | 1.90 (0.72–5.00) | 0.192 | 1.62 (0.63–4.15) | 0.318 |
| $200 000–$499 999 | 0.71 (0.45–1.12) | 0.137 | 1.00 (0.64–1.57) | 0.986 | 1.31 (0.53–3.21) | 0.560 | 1.59 (0.67–3.76) | 0.295 |
| Less than $200 000 | 0.92 (0.60–1.41) | 0.704 | 1.43 (0.93–2.18) | 0.101 | 1.95 (0.84–4.51) | 0.118 | 2.00 (0.89–4.51) | 0.094 |
| None | 0.89 (0.57–1.40) | 0.611 | 1.20 (0.77–1.87) | 0.427 | 2.58 (1.08–6.16) | 0.032 | 2.71 (1.17–6.32) | 0.021 |
| **Disruptions to healthy eating and sleep** |  |  |  |  |  |  |  |  |
| Low | 1.00 |  | 1.00 |  | 1.00 |  | 1.00 |  |
| Medium | 2.16 (1.57–2.96) | <0.001 | 2.41 (1.83–3.19) | <0.001 | 2.10 (1.09–4.05) | 0.027 | 2.14 (1.19–3.86) | 0.011 |
| High | 4.89 (3.51–6.80) | <0.001 | 7.42 (5.53–9.95) | <0.001 | 6.20 (3.09–12.43) | <0.001 | 5.82 (3.09–10.95) | <0.001 |
| **Disruptions to socializing and leisure activities** |  |  |  |  |  |  |  |  |
| Low | 1.00 |  | 1.00 |  | 1.00 |  | 1.00 |  |
| Medium | 1.64 (1.17–2.30) | 0.004 | 1.78 (1.34–2.37) | <0.001 | 1.97 (0.97–3.99) | 0.059 | 1.59 (0.88–2.88) | 0.121 |
| High | 3.97 (2.82–5.59) | <0.001 | 3.02 (2.24–4.07) | <0.001 | 4.91 (2.37–10.15) | <0.001 | 2.87 (1.55–5.32) | 0.001 |
| **Disruptions to household chores** |  |  |  |  |  |  |  |  |
| Low | NA |  | NA |  | 1.00 |  | 1.00 |  |
| Medium | NA |  | NA |  | 0.63 (0.42–0.96) | 0.032 | 1.01 (0.67–1.50) | 0.980 |
| High | NA |  | NA |  | 1.11 (0.65–1.89) | 0.696 | 1.35 (0.80–2.27) | 0.264 |
| **Disruptions to** **exercising or keeping active** |  |  |  |  |  |  |  |  |
| Low | NA |  | NA |  | 1.00 |  | 1.00 |  |
| Medium | NA |  | NA |  | 0.81 (0.51–1.29) | 0.377 | 0.73 (0.48–1.12) | 0.151 |
| High | NA |  | NA |  | 1.40 (0.85–2.32) | 0.186 | 1.47 (0.93–2.35) | 0.102 |
| **Disruptions to work or study** |  |  |  |  |  |  |  |  |
| Low | NA |  | NA |  | 1.00 |  | 1.00 |  |
| Medium | NA |  | NA |  | 1.32 (0.83–2.09) | 0.239 | 0.86 (0.56–1.31) | 0.470 |
| High | NA |  | NA |  | 2.12 (1.26–3.58) | 0.005 | 1.75 (1.08–2.84) | 0.022 |
| **Adoption of all preventive measures** |  |  |  |  |  |  |  |  |
| Yes | 1.00 |  | 1.00 |  | 1.00 |  | 1.00 |  |
| No | 0.99 (0.66–1.50) | 0.976 | 0.88 (0.61–1.26) | 0.481 | 1.17 (0.70–1.96) | 0.556 | 0.95 (0.58–1.57) | 0.846 |
| **Wear a mask when I go out** |  |  |  |  |  |  |  |  |
| Yes | 1.00 |  | 1.00 |  | 1.00 |  | 1.00 |  |
| No | 1.60 (0.84–3.07) | 0.155 | 1.68 (0.98–2.88) | 0.058 | 2.42 (0.43–13.78) | 0.318 | 0.97 (0.18–5.36) | 0.973 |
| **Wash hands often** |  |  |  |  |  |  |  |  |
| Yes | 1.00 |  | 1.00 |  | 1.00 |  | 1.00 |  |
| No | 1.03 (0.68–1.58) | 0.876 | 1.41 (1.00–1.99) | 0.047 | 0.99 (0.46–2.15) | 0.987 | 0.77 (0.37–1.64) | 0.504 |
| **Avoid people with respiratory symptoms** |  |  |  |  |  |  |  |  |
| Yes | 1.00 |  | 1.00 |  | 1.00 |  | 1.00 |  |
| No | 0.73 (0.55–0.97) | 0.033 | 1.10 (0.86–1.42) | 0.442 | 0.86 (0.50–1.48) | 0.582 | 0.96 (0.58–1.60) | 0.875 |
| **Avoid going to crowded places** |  |  |  |  |  |  |  |  |
| Yes | 1.00 |  | 1.00 |  | 1.00 |  | 1.00 |  |
| No | 0.89 (0.67–1.18) | 0.407 | 1.32 (1.03–1.68) | 0.026 | 1.12 (0.68–1.85) | 0.652 | 0.84 (0.52–1.38) | 0.492 |
| **Avoid using public transport** |  |  |  |  |  |  |  |  |
| Yes | 1.00 |  | 1.00 |  | 1.00 |  | 1.00 |  |
| No | 1.06 (0.74–1.53) | 0.742 | 1.07 (0.78–1.47) | 0.664 | 0.80 (0.50–1.27) | 0.337 | 0.81 (0.52–1.27) | 0.364 |
| **Stay at home as much as possible** |  |  |  |  |  |  |  |  |
| Yes | NA |  | NA |  | 1.00 |  | 1.00 |  |
| No | NA |  | NA |  | 1.26 (0.71–2.21) | 0.431 | 1.53 (0.90–2.58) | 0.113 |
| **Use hand sanitizer** |  |  |  |  |  |  |  |  |
| Yes | NA |  | NA |  | 1.00 |  | 1.00 |  |
| No | NA |  | NA |  | 1.05 (0.61–1.82) | 0.852 | 1.07 (0.65–1.78) | 0.789 |
| **Disinfect house** |  |  |  |  |  |  |  |  |
| Yes | NA |  | NA |  | 1.00 |  | 1.00 |  |
| No | NA |  | NA |  | 0.64 (0.39–1.03) | 0.065 | 0.95 (0.61–1.49) | 0.828 |
| **I worry about being infected with the COVID-19** |  |  |  |  |  |  |  |  |
| Not at all | 1.00 |  | 1.00 |  | NA |  | NA |  |
| Some/Quite/Very much | 2.47 (1.55–3.96) | <0.001 | 1.28 (0.92–1.77) | 0.141 | NA |  | NA |  |
| **If I get infected with the COVID-19, I can die from it** |  |  |  |  |  |  |  |  |
| Strongly disagree/Disagree | NA |  | NA |  | 1.00 |  | 1.00 |  |
| Strongly agree/Agree | NA |  | NA |  | 2.06 (1.15–3.68) | 0.015 | 1.78 (1.04–3.02) | 0.034 |
| **If I get infected with the COVID-19, it will cause serious long-term health problems** |  |  |  |  |  |  |  |  |
| Strongly disagree/Disagree | NA |  | NA |  | 1.00 |  | 1.00 |  |
| Strongly agree/Agree | NA |  | NA |  | 1.61 (0.64–4.05) | 0.311 | 2.05 (0.88–4.82) | 0.098 |
| **If I get infected with the COVID-19, the treatment side-effects will cause serious long-term health problems** |  |  |  |  |  |  |  |  |
| Strongly disagree/Disagree | NA |  | NA |  | 1.00 |  | 1.00 |  |
| Strongly agree/Agree | NA |  | NA |  | 0.50 (0.23–1.09) | 0.082 | 0.42 (0.21–0.86) | 0.017 |
| **The pandemic will cause a financial strain to me** |  |  |  |  |  |  |  |  |
| Strongly disagree/Disagree | NA |  | NA |  | 1.00 |  | 1.00 |  |
| Strongly agree/Agree | NA |  | NA |  | 2.10 (1.32–3.32) | 0.002 | 1.77 (1.16–2.71) | 0.008 |
| **The pandemic will reduce my income** |  |  |  |  |  |  |  |  |
| Strongly disagree/Disagree | NA |  | NA |  | 1.00 |  | 1.00 |  |
| Strongly agree/Agree | NA |  | NA |  | 1.01 (0.61–1.65) | 0.976 | 1.31 (0.83–2.08) | 0.247 |
| **The pandemic will reduce my savings** |  |  |  |  |  |  |  |  |
| Strongly disagree/Disagree | NA |  | NA |  | 1.00 |  | 1.00 |  |
| Strongly agree/Agree | NA |  | NA |  | 1.27 (0.76–2.10) | 0.361 | 1.07 (0.67–1.71) | 0.777 |

Data was weighted by gender, age, and education level based on the Hong Kong population census 2019. All dates are in 2020. **ǂ**The 7-item Generalized Anxiety Disorder scale (GAD-7) scores at or exceeding 10 were used to define clinical levels of anxiety symptoms. §The 9-item Patient Health Questionnaire (PHQ-9) scores at or exceeding 10 were used to define clinical levels of depressive symptoms. ¶Demographic variables that were not significant in bivariable analyses (Supplementary material 1) were not included in the multivariable logistic regression. NA=not applicable (question was not asked in the survey). COVID-19=coronavirus disease 2019.
